# Synthesis of biochar/MoS_2_ composite modified with poly(acrylic acid) (BC/MoS_2_/PAA) for the removal of Cd(ii) and Pb(ii) from wastewater

**DOI:** 10.1039/d5ra03405a

**Published:** 2025-08-26

**Authors:** Salami Hammed Olawale, Waleed Alahmad, Ibrahim A. Darwish, Mohammad Ashfaq, Ryhan J. Darling, Charoenkwan Kraiya

**Affiliations:** a Department of Chemistry, Faculty of Science, Chulalongkorn University Bangkok 10330 Thailand charoenkwan.k@chula.ac.th; b Electrochemistry and Optical Spectroscopy Center of Excellence, Department of Chemistry, Faculty of Science, Chulalongkorn University Bangkok 10330 Thailand; c Department of Pharmaceutical Chemistry, College of Pharmacy, King Saud University P. O. Box 2457 Riyadh 11451 Saudi Arabia; d Department of Biotechnology, School of Science, Woxsen University Hyderabad 502345 Telangana India mohdashfaqbiotech@gmail.com; e Department of Biochemistry, Medical College of Wisconsin Milwaukee WI 53226 USA

## Abstract

The present study focuses on the synthesis of coconut shell-derived biochar (BC), molybdenum disulfide (MoS_2_), and poly(acrylic acid) (PAA) (BC/MoS_2_/PAA) composite. The composite was synthesized *via* a simple hydrothermal method. The structural and morphological features of the resulting composite were thoroughly characterized using Fourier-transform infrared spectroscopy (FTIR), X-ray diffraction (XRD), scanning electron microscopy with energy-dispersive X-ray spectroscopy (SEM-EDS), Brunauer–Emmett–Teller (BET) surface analysis, and Raman spectroscopy. These analyses confirmed the successful formation and integration of the composite components. Adsorption isotherm studies revealed that Cd(ii) and Pb(ii) ions uptake by the BC/MoS_2_/PAA composite adhered to the Langmuir model, indicating monolayer adsorption onto a homogeneous surface. The maximum adsorption capacities for Cd(ii) and Pb(ii) were determined to be 8.23 mg g^−1^ and 26.47 mg g^−1^, respectively. Kinetic investigations indicated that the adsorption process followed a pseudo-second-order model, suggesting that chemisorption was the dominant mechanism. Moreover, the composite exhibited excellent reusability and selectivity towards Cd(ii) and Pb(ii) ions. Oxygen-containing functional groups, sulfide ions (S^2−^), and π–π interactions within the composite imply that electrostatic attraction, surface complexation, and cation–π interactions were the primary forces governing the adsorption process. These findings highlight the BC/MoS_2_/PAA composite's significant potential for effectively removing Cd(ii) and Pb(ii) from contaminated wastewater.

## Abstract 2

The present study focuses on the synthesis of coconut shell-derived biochar (BC), molybdenum disulfide (MoS_2_), and poly(acrylic acid) (PAA) (BC/MoS_2_/PAA) composite.

## Graphical abstract

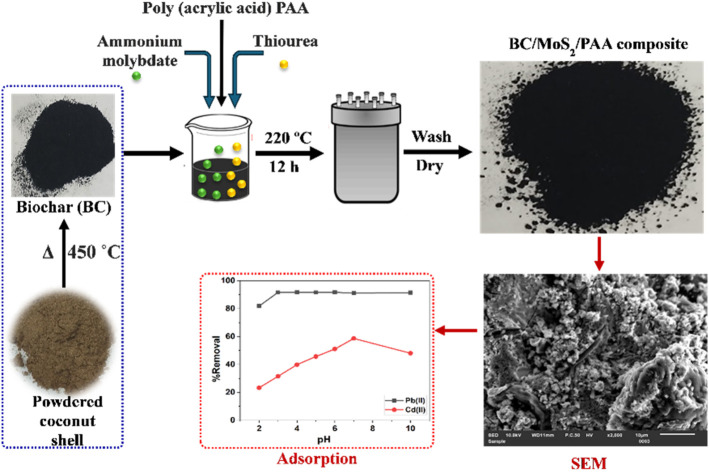

## 1. Introduction

Heavy metals' contamination of aquatic ecosystems has emerged as a pressing environmental concern due to their high toxicity even at trace concentrations, carcinogenic nature, and resistance to biodegradation. These properties make heavy metals particularly hazardous, posing serious threats to environmental sustainability and public health.^1^ Rapid urbanization and industrial growth have significantly contributed to the release of heavy metal ions such as Cd(ii) and Pb(ii) into aquatic environments, either directly or indirectly, from various sources, including metal plating, mining operations, tanneries, battery manufacturing, and the use of pesticides and fertilizers.^2^ Overexposure of Pb can lead to serious damage to the brain, kidneys, and reproductive system^3^ and cadmium toxicity has been linked to adverse health effects including hypertension, kidney dysfunction, and lung damage.^4^ According to the World Health Organization (WHO), the maximum permissible concentrations of Cd(ii) and Pb(ii) in drinking water are 0.003 mg L^−1^ and 0.05 mg L^−1^, respectively. Consequently, effectively removing these toxic metal ions from wastewater is essential to ensure environmental safety and public health.

Among various water treatment techniques, adsorption is widely recognized as one of the most effective methods due to its high removal efficiency, wide applicability across different contaminants, and minimal environmental footprint.^5^ Conventional adsorbents, including activated carbon, alumina, zeolites, and clays, have been extensively employed to remove heavy metal ions such as Cd(ii) and Pb(ii) from aqueous solution.^6^ However, these materials often show low adsorption capacity, weak metal ion binding, and poor selectivity.^7^ Therefore, selecting adsorbents with high selectivity and strong adsorption performance is essential for effective water purification.

There has been growing interest in eco-friendly adsorbent materials derived from agricultural byproducts and biomass waste, such as coconut shells, due to their low cost, wide availability, and ease of production.^5^ Among these adsorbents, biochar (BC) a carbon-rich material produced through the pyrolysis of biomass under limited oxygen conditions, has gained increasing attention due to its cost-effectiveness, environmental friendliness, and favorable physicochemical properties for heavy metal removal.^8^ It has emerged as a promising candidate for environmental remediation. Due to its high surface area, porous structure, and abundance of surface functional groups, biochar exhibits strong adsorption affinity for heavy metal ions, offering a cost-effective and efficient solution for removing contaminants from aqueous environments.^9^ Sakhiya *et al.*,^10^ reported 17.93 mg g^−1^ adsorption capacity for Pb(ii) using the prepared biochar from rice straw (300–500 °C). Many studies suggested an improved adsorption capacity of heavy metals with biochar surface modification as the adsorption performance of raw biochar is relatively low on heavy metal ions removal.^11^ Therefore, the functionalization of biochar with nanomaterials has become an essential strategy for developing advanced composites with enhanced adsorption performance. Currently, various nanomaterials, including Fe_3_O_4_, MnO_2_, SiO_2_, and molybdenum disulfide (MoS_2_), have been employed as adsorbents for heavy metal ion removal,^12^ owing to their exceptional surface properties, high reactivity, and strong metal-binding capabilities.^9^ Among these nanomaterials, MoS_2_ has attracted much interest in recent years for the removal of heavy metals from aquatic environments, primarily due to its rich sulfur structure, which exhibits excellent affinity towards heavy metals through *via* soft–soft Lewis acid–base interactions and large specific surface area.^11^ Pristine MoS_2_ showed a very poor saturated adsorption capacity of 6.24 mg g^−1^ for cadmium ions.^13^ MoS_2_-NSH synthesized by Bebaei *et al.*, showed adsorption capacity of 15.00 mg g^−1^ for Pb^2+^ ion.^14^ However, MoS_2_ suffers the challenge of poor dispersibility, self-aggregation, and layer restacking, which hinders its adsorption efficiency for Pb(ii) and Cd(ii) ions.^15^ However, one of the most effective strategies for improving adsorption capacity and decrease aggregation through improved dispersibility of MoS_2_ is through impregnation into BC^15^ as support to form BC/MoS_2_ and expansion of the interlayer spacing through inserting polymer chains between the layers^16^ such as polyvinyl alcohol (PVA),^17^ polyaniline,^18^ polydopamine^19^*etc.* Previously, Zhu, *et al.*,^20^ fabricate a bio-inspired composite aerogel consisting of MoS_2_ and RGO sheets modified with polydopamine PDA for the adsorption of methylene green, in which the PDA act as a dispersant to prevent aggregation and to strengthen interaction between MoS_2_ and RGO, thereby enhancing the adsorption capacity for removal of methylene green. Li *et al.*, introduced polyaniline between MoS_2_ and RGO layers for High-Performance Supercapacitors.^21^ Therefore, for the first time, the common aggregation that easily occur in MoS_2_ can be addressed by addition of poly(acrylic acid).

Poly(acrylic acid) (PAA), is a widely used, cost-effective, and biodegradable polymer characterized by a high density of carboxylic acid groups capable of forming stable coordination complexes with metal ions in aqueous solutions.^22^ PAA has demonstrated effectiveness in wastewater treatment applications, largely due to its hydrophilic nature, which enhances the dispersibility of materials in highly polar aqueous environments.^23^ Hence, the incorporation of PAA to the BC/MoS_2_ heterostructure surface will thus, improve the dispersibility of both BC and MoS_2_ to prevent aggregation and strengthen the interfacial bonding between the biochar (BC) and MoS_2_, leading to the exposure of more S atom active sites that will significantly enhance the removal efficiency of Pb(ii) and Cd(ii) and provides convenient handling and recyclability.^24^

In this work, biochar (BC) from waste biomass (coconut shell) was processed by a slow pyrolysis reaction, which was sustainably applied as template material modified with MoS_2_ to form heterostructure (BC/MoS_2_) incorporated with PAA (BC/MoS_2_/PAA) *via* one-pot hydrothermal method as an effective strategy implemented to improve the adsorption capacity of Cd(ii) and Pb(ii). The surface morphology and physicochemical properties of the BC/MoS_2_/PAA composite were comprehensively characterized using scanning electron microscopy (SEM), energy-dispersive X-ray spectroscopy (EDS), Fourier-transform infrared spectroscopy (FTIR), Raman spectroscopy, and X-ray diffraction (XRD) analysis. The adsorption performance was studied through the experiment of the adsorption kinetics, isotherm, dosage, and pH effect. The regeneration capability and competitive adsorption performance of the BC/MoS_2_/PAA composite were thoroughly evaluated. The results demonstrated that the composite exhibits excellent adsorption efficiency for removing Cd(ii) and Pb(ii) from aqueous solutions compared to previous conventional adsorbent.

## 2. Material and methods

### 2.1. Chemical and reagents

Thiourea (H_2_NCSNH_2_), ammonium molybdate (NH_4_)_6_Mo_7_O_24_·4H_2_O), poly(acrylic acid sodium salt, *M*_w_ = ∼5100), sodium chloride (NaCl), nitric acid (HNO_3_), hydrochloric acid (HCl), lead nitrate (Pb(NO_3_)_2_), and cadmium nitrate tetrahydrate (Cd(NO_3_)_2_·4H_2_O), magnesium nitrate hexahydrate (Mg(NO_3_)_2_·6H_2_O, cobalt(ii) acetate (Co(oAc)_2_), iron(iii) sulfate (Fe_2_(SO_4_)_3_), sodium nitrate (NaNO_3_), and copper(ii) acetate (Cu(oAc)_2_) were purchased from Sigma-Aldrich Co., Ltd. Coconut shell was purchased from local Khlong Toei market, Bangkok, Thailand. Milli-Q water was used throughout the experiment. All chemicals and reagents used in the experiments were of analytical grade and used without further purification.

### 2.2. Synthesis of coconut shell-based biochar (BC)

Coconut shells were purchased from Khlong Toei market, Bangkok, Thailand. The coconut shell-based BC was synthesized by pyrolysis method.^25^ Coconut shell samples were thoroughly washed with deionized water to remove surface impurities and then air-dried at room temperature for 2–3 days. The dried shells were subsequently ground into a fine powder and sieved through a 60-mesh (0.250 mm) screen. A 10 g portion of the sieved powder was placed in a covered crucible and subjected to pyrolysis in a muffle furnace. The heating was carried out at a rate of 20 °C per minute until reaching 450 °C, where the sample was maintained for 2 hours. After natural cooling to room temperature, the resulting black powder—biochar—was labelled as BC and stored in an airtight container for subsequent use. The photograph of synthesized coconut shell-based BC is shown in Fig. S1 (see SI).

#### 2.2.1. Preparation of BC/MoS_2_ composite

The BC/MoS_2_ composite was synthesized using a straightforward hydrothermal method. In this process, 0.50 g of the synthesized BC was dispersed in 60 mL of deionized (DI) water and subjected to sonication for 30 minutes to form a homogeneous dark suspension. Subsequently, 1.15 g of ammonium heptamolybdate tetrahydrate ((NH_4_)_6_Mo_7_O_24_·4H_2_O) and 2.30 g of thiourea (H_2_NCSNH_2_), serving as the Mo and S precursors, respectively, were added to the suspension. The mixture was stirred for 30 minutes using a magnetic stirrer. Following this, the suspension was transferred to a 100 mL Teflon-lined stainless-steel autoclave and heated at 220 °C for 12 hours. After the reaction was complete and the system cooled, a black precipitate was collected, filtered, and washed thoroughly with deionized water three times before freeze-drying. The resulting BC/MoS_2_ composite was stored in a desiccator for further characterization and use.

#### 2.2.2. Preparation of BC/MoS_2_/PAA composite

In the synthesis, 0.50 gram of synthesized BC and 0.50 gram of PAA were mixed into 60 mL of deionized (DI) water, followed by sonication treatment for 30 min to form a dark suspension. Subsequently, 0.575 g of ammonium heptamolybdate tetrahydrate ((NH_4_)_6_Mo_7_O_24_·4H_2_O) and 1.15 g of thiourea (H_2_NCSNH_2_), as the sources of Mo and S, respectively, were added to the suspension and stirred for 30 minutes using a magnetic stirrer. Following the stirring process, the suspension was transferred to a 100 mL Teflon-lined stainless-steel autoclave, which was then heated to 220 °C for 12 hours. After the reaction, the system was allowed to cool, and a black precipitate was collected. This precipitate was then filtered, washed thoroughly with deionized water three times, and freeze-dried. The obtained BC/MoS_2_/PAA composite was stored in a desiccator before use. The pictures of synthesized BC/MoS_2_/PAA composite is shown in SI (Fig. S1c).

### 2.3. Characterization of prepared BC/MoS_2_/PAA composite

The surface morphologies and the distribution of surface elements of the synthesized composite were evaluated by an SEM equipped with an EDS (JEOL IT-300). An INVIA Raman microscope equipped with a 514 nm Ar laser (Renishaw, UK) was used to obtain the Raman spectra. Fourier-transform infrared (FTIR) spectroscopy was performed using a Thermo Fisher Scientific Nicolet IS50 spectrometer, with spectra recorded in the 4000–400 cm^−1^ range using KBr pellets. The crystalline phase of the powders was analyzed by X-ray diffraction (XRD) on a Bruker D8 Advance X-ray diffractometer equipped with a graphite monochromatized Cu Kα radiation source, operating at 35 mA and 40 kV. The composite's Brunauer–Emmett–Teller (BET) surface area was determined at 77 K using a BELSORP-mini surface area analyzer. The concentrations of Cd(ii) and Pb(ii) were quantified using an inductively coupled plasma-optical emission spectrometer (ICP-OES) (Thermo Fisher Scientific, ICAP DUO 6500).

### 2.4. pH of point of zero charge (pH_pzc_)

Peer *et al.*^26^ used a salt method to evaluate the pH of point of zero charge (pH_pzc_). Briefly, a 0.01 M NaCl solution was prepared and added into 60 mL polyethylene bottles with adjusted pH using 0.1 M HCl and NaOH solution in the range from 2 to 9. Then, in each 10 mL solution, 0.03 g of BC/MoS_2_/PAA adsorbent was added and shaken for 24 hour. Thereafter, the pH_pzc_ of the synthesized adsorbent was determined by calculating the difference between the final and initial pH values, with the point of intersection on the *x*-axis in the curve recorded as the pH_pzc_.

### 2.5. Batch adsorption

Batch adsorption experiments were performed in 60 mL polyethylene bottles, where 0.01 g of BC/MoS_2_/PAA composite was added to 10 mL of 10 mg L^−1^ Cd(ii) and Pb(ii) solutions, which were prepared using Pb(NO_3_)_2_ and Cd(NO_3_)_2_·4H_2_O as the metal sources. The bottles were agitated on a shaker at 30 rpm and maintained at room temperature for 720 minutes. After the adsorption period, the solutions were filtered through a 0.22 μm filter, and the remaining concentrations of Cd(ii) and Pb(ii) in the filtrates were measured using ICP-OES. The effect of adsorbent dosage, pH, contact times, and initial concentrations were evaluated as the optimized parameters. The effect of various dosages of adsorbents (0.005, 0.010, 0.015, 0.020, and 0.025 g) was studied. The effect of pH on Cd(ii) and Pb(ii) adsorption was studied by adjusting initial pH values using 0.1 M HNO_3_ and 0.1 M NaOH solution from 2 to 9. The effect of contact time in this study was evaluated at different time intervals between 15 to 1080 min, while the effect of initial Cd(ii) and Pb(ii) concentrations was evaluated from 10 mg L^−1^ to 30 mg L^−1^ for Pb(ii) and 1 mg L^−1^ to 20 mg L^−1^ for Cd(ii) were assessed.

Each measurement was conducted in triplicate using the ICP-OES. The equilibrium adsorption capacity and the removal efficiency (%) for Cd(ii) and Pb(ii) were calculated according to the following equations:1
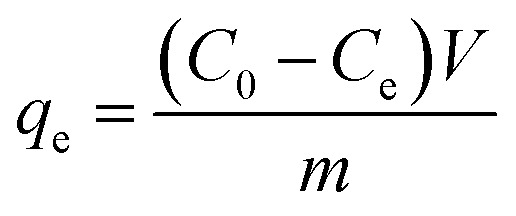
2
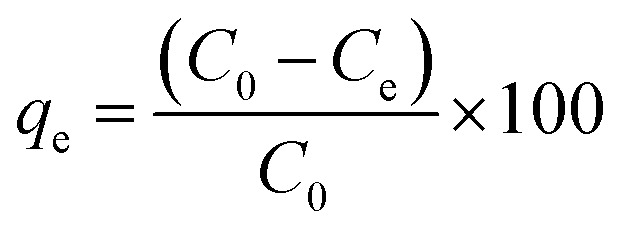
where *C*_0_ and *C*_e_ are initial and equilibrium concentrations (mg L^−1^), respectively; *m* is the mass of the adsorbent (g) used in the experiment; and *V* is the volume of the solution (L).

The adsorption kinetics significantly investigates the relationship between adsorption time, adsorption capacity at equilibrium, and concentration. Three kinetic models, pseudo-first-order, pseudo-second order, and intraparticle diffusion models, were considered to evaluate Cd(ii) and Pb(ii) adsorption processes onto BC/MoS_2_/PAA. The equation for each model was expressed in eqn (3)–(5), and the plot of ln(*q*_e_ − *q*_*t*_) *versus t*, *t*/*q*_*t*_*versus t*, and *q*_*t*_*versus t*^0.5^ should be a straight line, respectively.^27^

Pseudo-first-order model equation

3 ln(*q*_e_ − *q*_*t*_) = ln *q*_e_ − *k*_1_*t*

Pseudo-second-order model equation,4
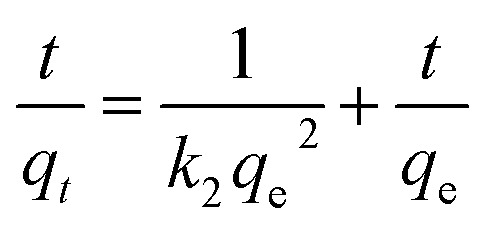


Intraparticle diffusion model equation

5 *q*_*t*_ = *k*_id_*t*^0.5^ + *C*_i_

The adsorption capacity of Cd(ii) and Pb(ii) on BC/MoS_2_/PAA at equilibrium and at time *t*, respectively, is denoted by *q*_e_ and *q*_*t*_; the pseudo-first order and pseudo-second-order rate constants are denoted by *k*_1_ (min^−1^) and *k*_2_ (mg g^−1^ min^−1^), respectively. *C* is a constant proportional to the thickness of the boundary layer, mg g^−1^, and *k*_id_ is the intraparticle diffusion rate constant (mg g^−1^ min^−0.5^).

To understand the distribution and mechanism of Cd(ii) and Pb(ii) onto the BC/MoS_2_/PAA composite when the adsorption process attained equilibrium, the adsorption isotherm model plays a vital role. Therefore, three adsorption isotherm models such as Langmuir, Freundlich, and Temkin isotherm model, respectively with equations expressed^27^ in eqn (6)–(8) were considered to analyze the adsorption processes of Cd(ii) and Pb(ii) onto BC/MoS_2_/PAA.6
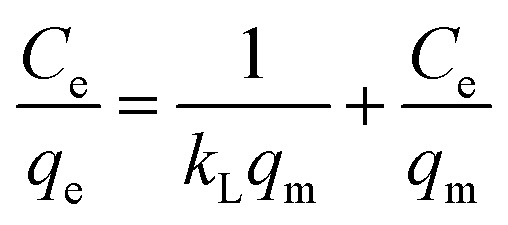

7 ln   *q*_e_ = ln *K*_F_ + 1/*n* ln *C*_e_

8 *q*_e_ = *B*_T_ ln *K*_T_ + *B*_T_ ln *C*_e_

In the equation, *q*_e_ is the adsorption capacity at equilibrium concentration (mg g^−1^), *q*_m_ is the maximum adsorption capacity (mg g^−1^) from Langmuir model, *C*_e_ is the equilibrium concentration of Pb(ii) (mg L^−1^), *K*_L_, *K*_F,_ and *K*_T_ represents the Langmuir constant (L mg^−1^), Freundlich constant (mg g^−1^), and Temkin constant (L g^−1^), respectively, *n* is a constant, which represents the sorption intensity, *B*_T_ is constant related to sorption heat (J mol^−1^).

## 3. Result and discussion

### 3.1. pH of point of zero charge (pH_pzc_) of BC/MoS_2_/PAA

**Fig. 1:**
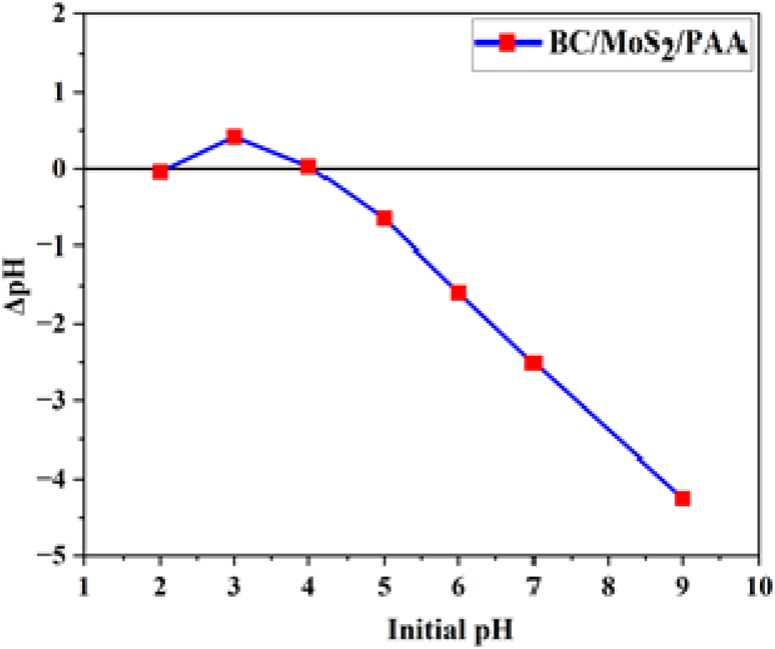
Point of zero charge (pH_pzc_) of BC/MoS_2_/PAA adsorbent.

The pH of the point of zero charge (pH_pzc_) is an important characteristic that reveals the surface charge component of an adsorbent, which indicates the pH point at which the positive and negative charge are of equal amount.^28^ In Fig. 1, the result of plot shows that pH_pzc_ value of BC/MoS_2_/PAA composite was found to be 4.10, suggesting a positive charge adsorbent surface at pH below the pH_pzc_ as pH decreases and negative charge adsorbent surface as the pH increases, which is suitable for the removal of positively charged ions such as Cd(ii) and Pb(ii) through electrostatic interaction as a possible mechanism involve in the adsorption process.^29^

### 3.2. Characterization

#### 3.2.1. SEM and EDS analysis

**Fig. 2:**
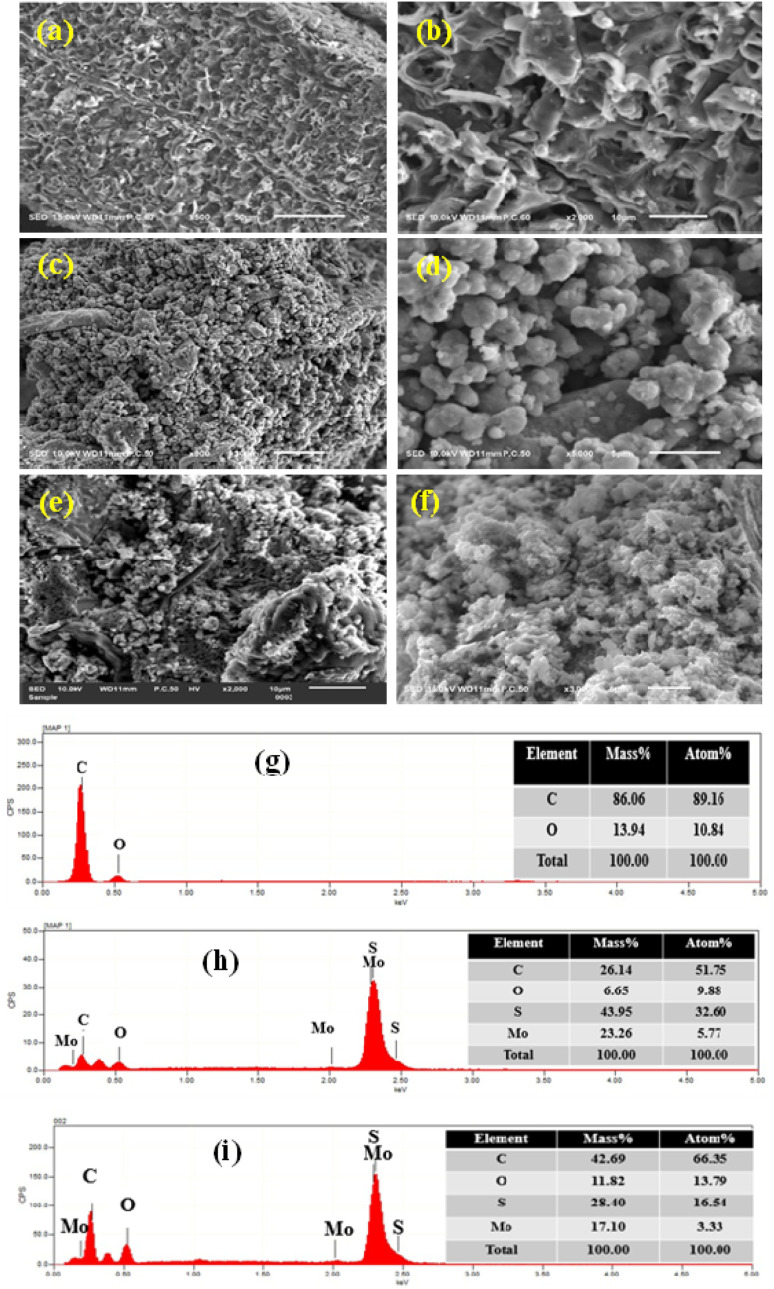
SEM image of (a and b) BC, (c and d) BC/MoS_2_, and (e and f) BC/MoS_2_/PAA. EDS analysis of (g) BC, (h) BC/MoS_2_, and (i) BC/MoS_2_/PAA composite.

In Fig. 2, the results of surface morphologies of the synthesized composite are presented. In Fig. 2(a and b), the SEM observation of BC showed a fish net-like amorphous structure that is not uniform with different sizes of pore diameter. For BC/MoS_2_ in Fig. 2(c and d), a homogeneous contact between BC and MoS_2_ layered with agglomerated rocky-like structure was observed. In Fig. 2(e and f), a dispersed, rough and coarse surface was observed when PAA was incorporated indicating that the BC was uniformly coated by the polymer matrix, thereby providing an excellent surface for the growth of MoS_2_. In Fig. 2(g–i), the EDS spectrum shows the presence of carbon, oxygen, molybdenum, and sulphur in the BC, BC/MoS_2_, and BC/MoS_2_/PAA composite in different atom and mass% of each element.

#### 3.2.2. XRD analysis

**Fig. 3:**
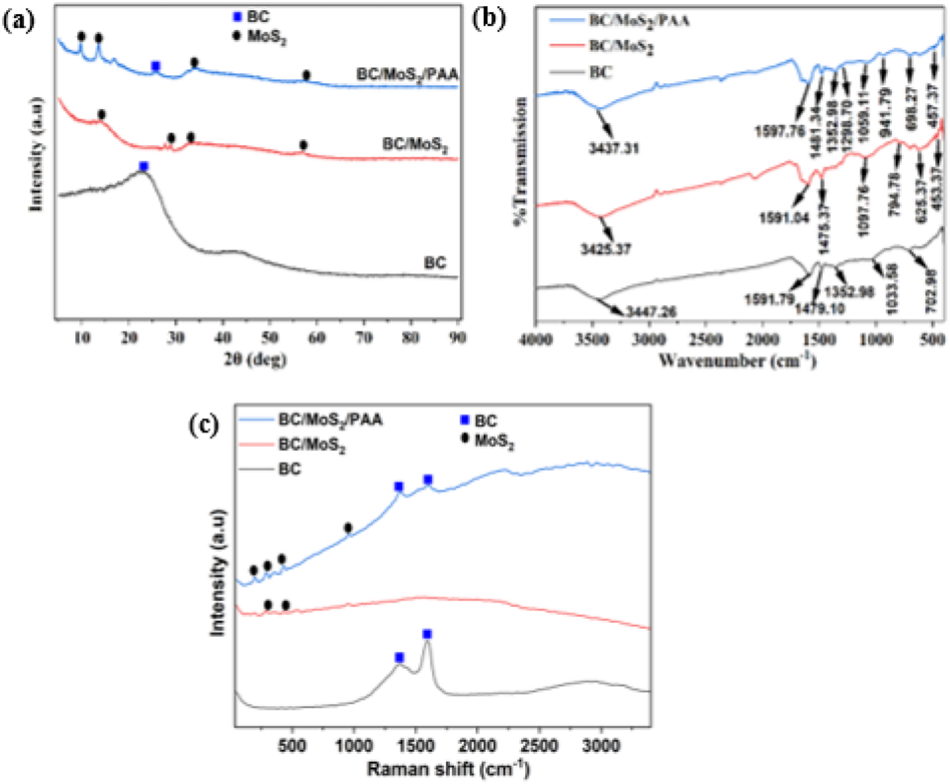
(a) XRD, (b) FTIR, and (c) Raman spectra of the BC, BC/MoS_2,_ and BC/MoS_2_/PAA composite.

The crystalline phase of the as-prepared composite was investigated by XRD (Fig. 3a). In BC, the broad peak at 22.86° and 43.68° correspond to the characteristic peak of carbon material with an amorphous structure, indicating that the BC contains lignin or cellulose after pyrolysis of the coconut shell feedstock.^30^ In the BC/MoS_2_ crystal structure, a broad new peak at 14.14° could be assigned to the 002 planes of MoS_2_ nanosheet.^11^ It was observed that the characteristic peak of BC becomes embedded in the MoS_2_ sheet, indicating an intimate contact between BC and MoS_2_. With the incorporation of PAA, two new diffraction peaks emerged at the low-angle region of about 9.7° and 13.6° corresponding to 002 and 004 planes due to interlayer distance (9.09 and 6.52 Å)^31^ of MoS_2_ nanosheet. The enlarged interlayer spacing of 9.09 Å suggests the value for the existence of carbon layer in the interlayers of MoS_2_ generated by the *in situ* thermal decomposition of PAA.^32^ At 25.82°, a less intense peak of BC re-appeared, indicating uniform dispersibility of PAA and enhanced interlayer spacing thereby supporting the growth of MoS_2_ on the BC surface, suggesting the successful synthesis of BC/MoS_2_/PAA composite.

#### 3.2.3. FTIR analysis

In Fig. 3b, the result shows the characteristics peak of BC, BC/MoS_2,_ and BC/MoS_2_/PAA composite as evaluated from the FTIR spectra. In the BC spectra, a broad peak at 3447.26 cm^−1^ correspond to the –OH bond stretching vibration.^33^ At 1591.04 cm^−1^ and 1475.37 cm^−1^, the peak represent the presence of C=C and COO^−^ in the aromatic ring and phenolic group.^33,34^ The weak peak observed at 1052.98 cm^−1^ and 702.98 cm^−1^ is attributed to C–O bond and C–H bond in coconut shell-derived biochar.^35^ In the BC/MoS_2_ spectra, the peak observed at approximately 2350 cm^−1^ is attributed to the stretching and bending of O–Mo vibrations, indicating the participation of oxygen–molybdenum interaction in the sample.^36^ At 794.78 and 625.37 cm^−1^, the peak indicating the presence of aromatic –C–H bond^37^ and –SH bond on MoS_2_ nanosheet^36^ while the peaks observed at 453.37 cm^−1^ are attributed to Mo–S bonding vibration, indicating the successful loading of MoS_2_ on the biochar surface.^11^ A new peak at 1352.98 and 941.79 cm^−1^ attributing to C–OH stretching vibration^38^ and C–S deformation^39^ were observed after the incorporation of PAA. Additionally, the absence of distinct PAA peaks suggests its uniform dispersion within the composite matrix, suggesting the successful synthesis of BC/MoS_2_/PAA composite.

#### 3.2.4. Raman analysis

The Raman spectra was further used to characterize BC, BC/MoS_2,_ and BC/MoS_2_/PAA composites as shown in Fig. 3c. In the BC spectra, two strong peaks at 1368.72 and 1592.64 cm^−1^ corresponding to D-band and G-band, which are related to amorphous carbon and graphitic carbon respectively.^11^ The intensity ratio (*I*_D_/*I*_G_) obtained was 0.73, indicating the degree of graphitization of the BC. In BC/MoS_2_ spectra, two less intense peaks were detected at 302.86, and 436.09 cm^−1^ corresponding to the characteristic peaks of MoS_2_, and are attributed to the Mo and S atom (in-plane and out-of-plane vibration of Mo and S).^40^ Furthermore, no characteristic peaks of BC were observed, owing to the good interlacing of BC on the MoS_2_ surfaces, which is like the XRD result (Fig. 3a). After the incorporation of PAA, intense characteristic peaks of MoS_2_ were observed with new peaks at 201.72 and 953.67 cm^−1^, indicating doublet, suggesting that MoS_2_ is possibly undergoing partial oxidation or due to interlayer spacing,^40^ which is similar to the result of FTIR (Fig. 3b). The degree of graphitization of BC (*I*_D_/*I*_G_) also increases to 0.97. Hence, the PAA has been shown to be a dispersing agent that support the uniform coating and growth of MoS_2_ through formation of large interlayer spacing, as evident in XRD and FTIR spectra (Fig. 3a and b), indicating the successful synthesis of BC/MoS_2_/PAA composite.

#### 3.2.5. N_2_ adsorption–desorption isotherm

**Fig. 4:**
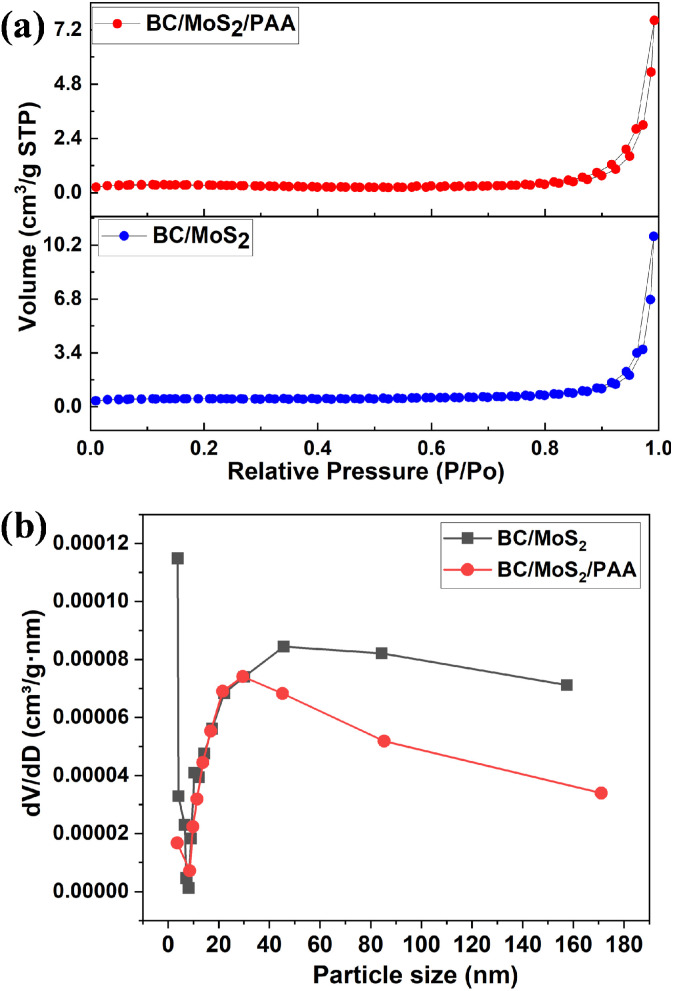
Linearized N_2_ adsorption–desorption isotherms and pore size distributions for the (a) BC/MoS_2_ and (b) BC/MoS_2_/PAA composite.

In Fig. 4, the BET surface area and pore-size distribution of BC/MoS_2_ and BC/MoS_2_/PAA composite were determined. The N_2_ adsorption/desorption isotherm curve of BC/MoS_2_ and BC/MoS_2_/PAA exhibited a type II shape, which is typical characteristic of macroporous materials and the BET-specific surface area and BJH for BC/MoS_2_ was 1.42 m^2^ g^−1^ and 58.6896 nm. After modification with PAA, the BET-specific surface area and BJH for BC/MoS_2_/PAA was 0.86 m^2^ g^−1^ and 52.6647 nm. Following the modification with PAA, the composite surface area decreases, indicating the deposition of PAA into the biochar's pore structure, which result in blocking of the pore channels.^41^ Therefore, these demonstrated that the lower surface area of the composite is a less dominant factor to be considered in explaining the mechanism governing the adsorption of pollutants. Hence, the macroporous nature of the composite with large pore size provides improved accessibility of the metal ion to the active sites and the large interlayer spacing of MoS_2_ as confirmed from the XRD result, support the exposure of the S^2−^ active sites for easy access by the heavy metals, thereby resulting in increase of the removal efficiency.

### 3.3. Adsorption studies

#### 3.3.1. Sorbent formulation

**Fig. 5:**
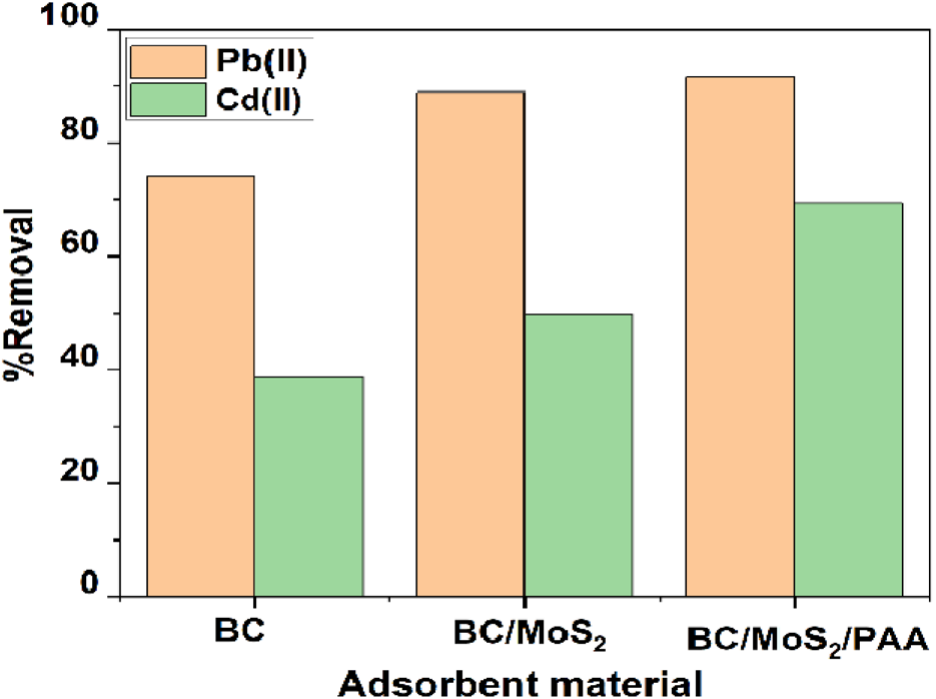
Comparison of percentage removal of Pb(ii) and Cd(ii) on adsorbent material (experimental conditions: concentration = 10 mg L^−1^; dose = 0.010 g/10 mL; time = 720 min).

From Fig. 5, the screening test for the adsorbents material was carried out to evaluate the adsorption percentage of each composite in the removal of Pb(ii) and Cd(ii) respectively. For the BC adsorbent, the adsorption percentages of Pb(ii) and Cd(ii) were 74.14 and 38.89%. When BC is modified with MoS_2_ with mass ratio of 0.5 : 1 : 2 for BC : Mo : S, there was an increase in adsorption percentage to 88.90 and 49.82% for Pb(ii) and Cd(ii). Furthermore, with the incorporation of PAA to form BC/MoS_2_/PAA composite, leads to higher removal percentage of Pb(ii) and Cd(ii) to 91.27 and 69.27% respectively. The best mass ratio selected was 0.25 : 0.58 : 1.15 : 0.5 for BC : Mo : S : PAA, indicating that the presence of PAA contributed significantly to the removal percentage of Pb(ii) and Cd(ii).

#### 3.3.2. Influence of adsorbent dosage

**Fig. 6:**
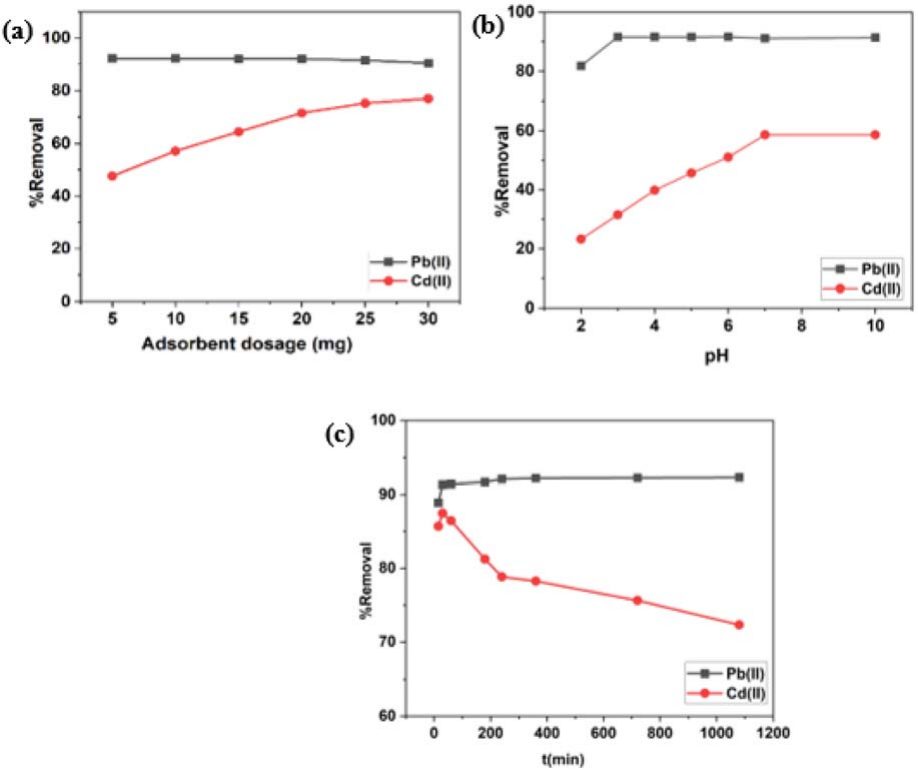
(a) Effect of adsorbent dosage; (b) effects of solution pH; (c) effects of contact time.

Fig. 6a shows the removal percentage of Pb(ii) and Cd(ii) at different doses of BC/MoS_2_/PAA from 0.005 g to 0.025 g. It was observed that within the range of 0.005–0.030 g adsorbent dosage, there is a slight decrease in the removal percentage from 92.23% to 90.37% for Pb(ii) as the adsorbent dosage increases. The decrease in removal percentage at high adsorbent dosage is due to aggregation of active sites of the adsorbent, which results in the decrease of total adsorption surface area with increased path length of diffusion^42,43^ while for Cd(ii), the percentage removal increases as the adsorbent dosage increase from 47.58% at 0.005 g up to 76.98% at 0.030 g. This can be attributed to the availability of more adsorption sites as the BC/MoS_2_/PAA dosage increases.^43^ Therefore, considering the economic cost and adsorption performance of BC/MoS_2_/PAA, the optimal adsorbent dosage of 0.010 g was selected for removing Pb(ii) and 0.025 g for Cd(ii) in wastewater.

#### 3.3.3. Effect of pH

The pH have significant influence on the adsorption of heavy metals. From Fig. 6b, the removal percentage of Pb(ii) and Cd(ii) onto BC/MoS_2_/PAA composite at various pH from 2 to 10 was studied. Based on the pH of point of zero charge of the BC/MoS_2_/PAA composite (pH_pzc_) in Fig. 1, the surface charge of the adsorbent will be negatively charged above the pH_pzc_, hence, having a high affinity for the adsorption of positively charged adsorbates. From Fig. 6b, it was observed that the percentage removal for Pb(ii) at pH 2 to pH 3, increases from 81.81% to 91.61%. From pH 3 to 6, the removal percentage was almost steady due to saturation of the surface with Pb^2+^ but slightly decreased from pH 7 to 10 due to repulsive electrostatic force that occur as a result of increase in concentration of Pb(OH)_3_^−^, and Pb(OH)_4_^2−^ formation with the negatively charge adsorption site at higher pH. For Cd(ii), the removal percentages sharply increase from 23.23% at pH 2 to 58.61% at pH 7, which is due to deprotonation of the active sites making the surface available for Cd(ii) adsorption as pH increases.^44^ From pH 7 to 10, the removal percentage remain almost steady due to saturation of the surface with Cd(ii).^44^ The generation of precipitation species such as Pb(OH)_2_ and Cd(OH)_2_ and adsorption of Pb^2+^ and Cd^2+^ ions are responsible for the decrease or increase in adsorption efficiency at higher pH.^45^ At lower pH value, there is usually a high concentration of proton which compete with the adsorbate for the adsorption site on the surface of the BC/MoS_2_/PAA composite thereby causing a decrease in adsorption efficiency while at a higher pH value, there is less concentration of proton species that results in less competition for adsorption site with the adsorbate, thereby resulting to increase in the adsorption efficiency.^45^ Therefore, the optimal pH selected for Cd(ii) and Pb(ii) removal for further experiments were pH 6 for Pb(ii) and pH 7 for Cd(ii).

#### 3.3.4. Effect of contact time

The equilibrium contact time is an important factor evaluated to determine the time of maximum sorption efficiency and the adsorption kinetics of the adsorbent. From Fig. 6c, various shaking durations ranging from 15, 30, 60, 180, 240, 360, 720 and 1080 min was investigated. As seen in Fig. 6c, during the first initial 30 min of contact, a high removal percentage of Pb(ii) and Cd(ii) was observed at 91.32% and 87.34%, due to the availability of a rich binding site on the BC/MoS_2_/PAA composite, thereby leading to the rapid sorption of Cd(ii) and Pb(ii). As the reaction progresses, no significant change occurred after 30 min to 240 min for Pb(ii) with a slight increase to 92.07% and remain almost the same until 1080 min, indicating that the adsorption system has reached equilibrium at 30 min while the removal percentage for Cd(ii) decreased drastically as contact time increases after 30 min until 1080 min, due to saturation of the binding sites, thereby increasing the steric hindrance that causes a decrease in active sites.^46^ Therefore, the contact time selected for both Cd(ii) and Pb(ii) was 30 min, which was used for further experiments.

### 3.4. Kinetic studies

**Fig. 7:**
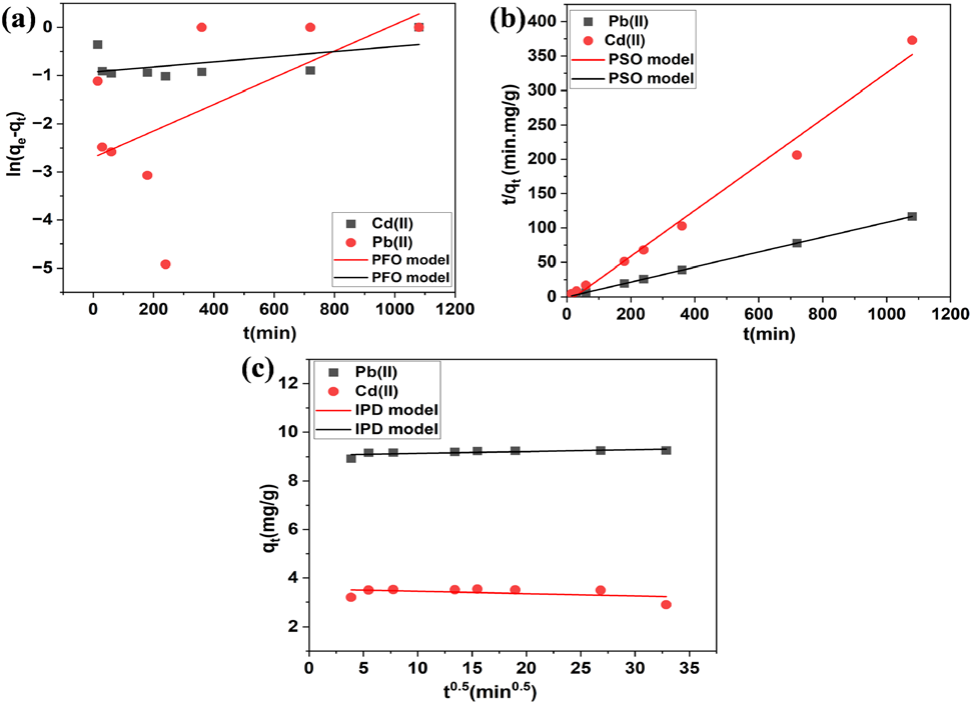
Linear plots of adsorption kinetics of Cd(ii) and Pb(ii) on BC/MoS_2_/PAA. (a) Pseudo-second order (b) pseudo-second order (c) intraparticle diffusion model (condition: concentration = 10 mg L^−1^; pH 6 for Pb(ii); time = 30 min; dosage = 0.010 gram and pH 7 for Cd(ii); adsorbent dosage = 0.025 gram).

Table 1 and Fig. 7a–c display the kinetic model data and the fitting curve of the kinetic model. As shown in Table 1, the correlation coefficient (*R*^2^) values obtained from the linear pseudo-second-order kinetic model were 1.0000 and 0.9882 for Pb(ii) and Cd(ii), which is higher than those of the pseudo-first-order kinetic and intra-particle diffusion model, and the experimental adsorption capacity (*q*_e,(exp)_ = 9.25 and 3.88 mg g^−1^) was closed to the theoretical value (*q*_e,(cal)_ = 9.23 and 3.42 mg g^−1^), indicating that the process of Cd(ii) and Pb(ii) adsorption on BC/MoS_2_/PAA is mainly controlled by chemisorption.^47^ From the non-linear pseudo-second order kinetic model as shown in Fig. S2 and Table S1, the value of *q*_e(cal)_ and *k*_2_ are obtained, where *k*_2_ for Pb(ii) is 0.21 g mg^−1^ min^−1^ and that of Cd(ii) is 0.61 g mg^−1^ min^−1^, indicating that the adsorption of Cd(ii) on BC/MoS_2_/PAA was faster than that of Pb(ii).

**Table 1:** Parameters calculated from the kinetic models

Kinetic model	Parameter	Pb(ii)	Cd(ii)
Pseudo-first order	*q*_e(exp)_ (mg g^−1^)	9.25	3.88
	*q*_e(cal)_ (mg g^−1^)	9.23	3.42
	*k*_1_ (1/min)	0.23	0.19
	*R* ^2^	0.3383	0.1841
Pseudo-second order	*q*_e(exp)_ (mg g^−1^)	9.25	3.88
	*q*_e(cal)_ (mg g^−1^)	9.27	3.42
	*k*_2_ (g mg^−1^ min^−1^)	0.21	0.61
	*R* ^2^	1.000	0.9882
Intraparticle diffusion	*k*_id_ (g mg^−1^ min^−0.5^)	0.062	2.66 × 10^−14^
	*C* (mg g^−1^)	8.94	3.39
	*R* ^2^	0.4927	0.9527

In Fig. 7c, the plot of *q*_*t*_*versus t*^0.5^ for the intraparticle diffusion (IPD) model shows that the curves did not pass through the origin, and intercept C reached a high level of 3.39 and 8.94 mg g^−1^ for Cd(ii) and Pb(ii) respectively. This indicates that intraparticle diffusion is not the rate-limiting step in adsorption.

### 3.5. Effect of initial concentration

**Fig. 8:**
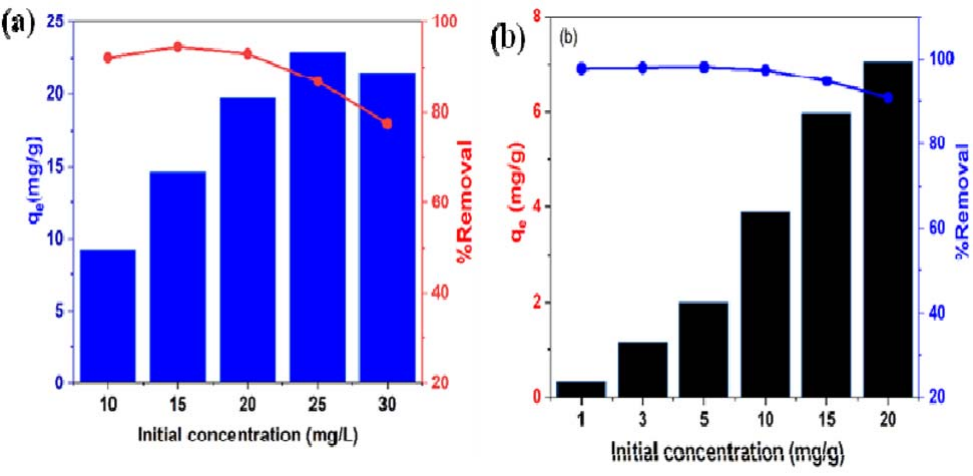
Effects of initial concentration on the adsorption of (a) Pb(ii) and Cd(ii) onto BC/MoS_2_/PAA (condition: pH 6 for Pb(ii); adsorbent dosage = 0.010 g; time = 30 min for Pb(ii) and pH 7 for Cd(ii); adsorbent dosage = 0.025 g; time = 30 min).

Effective adsorption is considered through the effect of initial concentration as an important factor, which is a function of the rate of adsorption. The result of adsorption capacities at different initial concentrations between 10–30 mg L^−1^ for Pb(ii) and 1–20 mg L^−1^ for Cd(ii) using BC/MoS_2_/PAA was reported in Fig. 8, which showed that adsorption capacity increases as the initial concentrations increase up to a certain threshold. This was due to the increased collision probability between the active sites of the adsorbent and the adsorbate. At higher initial concentrations of 30 mg L^−1^ for Pb(ii) and 20 mg L^−1^ for Cd(ii), saturation of the active sites tends to occur, resulting in competition by excess metal ions for the available binding sites, thereby leading to a decrease in percentage removal.^48^ To fully utilize the BC/MoS_2_/PAA composite before saturation of the active site occurs, the optimal initial concentration selected for Cd(ii) and Pb(ii) was 15 mg L^−1^ for Pb(ii) and 10 mg L^−1^ for Cd(ii) for further experiments.

### 3.6. Adsorption isotherm

**Fig. 9:**
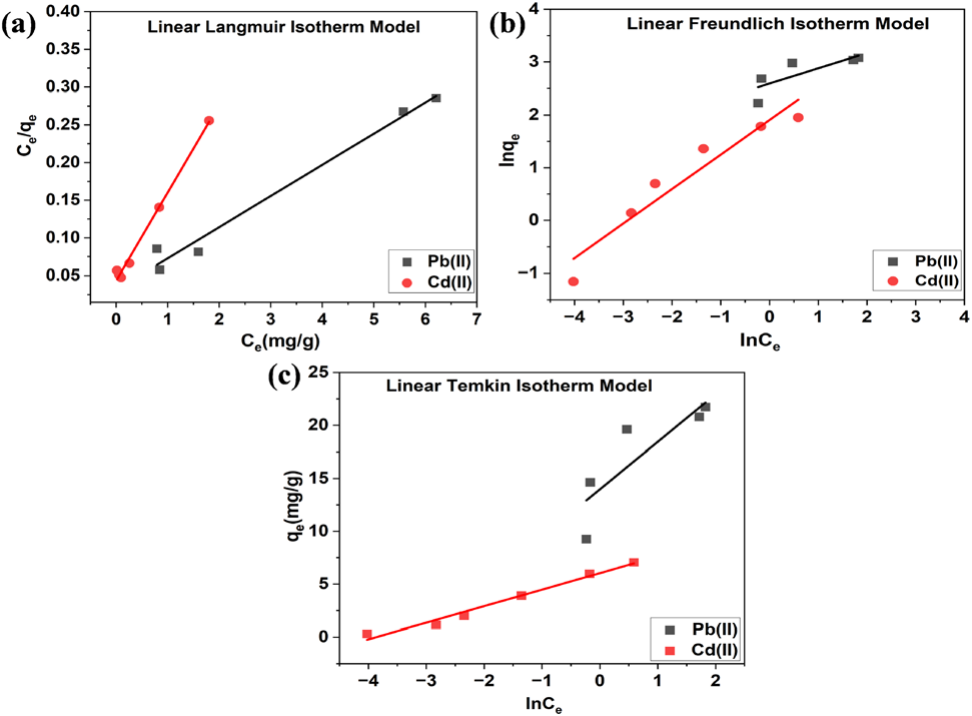
Adsorption isotherm model analysis. Linear plot for (a) Langmuir isotherm model (b) Freundlich isotherm model (c) Temkin isotherm model for Cd(ii) and Pb(ii) adsorption on BC/MoS_2_/PAA composite (condition: pH 6 for Pb(ii); dosage = 0.010 g; time = 30 min for Pb(ii) and pH 7 for Cd(ii); dosage = 0.025 g; time = 30 min).

Fig. 9(a–c) and Table 2 displays the fitting curve and experimental data obtained for the Langmuir, Freundlich, and Temkin isotherm model. For Pb(ii) and Cd(ii), the experimental data follows the Langmuir isotherm model (Fig. 9a), with a higher correlation coefficient (*R*^2^) of 0.9832 and 0.9914 compared to the Freundlich (0.6540 and 0.9232) and Temkin isotherm (0.7341 and 0.9832) model as obtained from the linear curve, suggesting a monolayer adsorption of Pb(ii) and Cd(ii) on homogenous surface of the BC/MoS_2_/PAA composite with uniform energies. Furthermore, the adsorption system can be investigated from the Langmuir model using the dimensionless parameter (*R*_L_), which is defined by the equation below:9
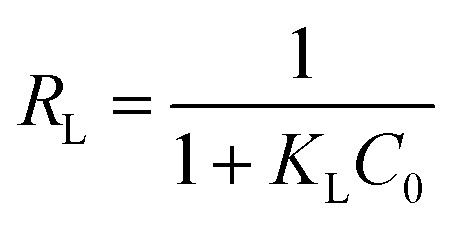
where *C*_0_ is the initial concentration of Pb(ii) and Cd(ii), *K*_L_ is the Langmuir constant. The value of *R*_L_ is the separation factor ranging between 0 and 1. Hence, the *R*_L_ value is applied to indicate the favorability of the adsorption process, which can be evaluated as irreversible (*R*_L_ = 0), favorable (0 < *R*_L_ < 1), linear (*R*_L_ = 1), and unfavorable (*R*_L_ > 1).^49^ Therefore, the *R*_L_ values obtained in the adsorption of Pb(ii) and Cd(ii) are between 0 and 1, indicating favorable adsorption by the BC/MoS_2_/PAA composite. In addition, from Freundlich isotherm data, the values of 1/*n* for the adsorption of Pb(ii) and Cd(ii) were less than one, suggesting a high affinity for the Pb(ii) and Cd(ii) adsorption by the BC/MoS_2_/PAA composite. From Fig. S3(a), the maximum adsorption capacity (*q*_max_) for Pb(ii) and Cd(ii) on the BC/MoS_2_/PAA composite obtained from the non-linear curve were 26.47 and 8.23 mg g^−1^.

**Table 2:** Fitting experimental data from the Linear Langmuir, Freundlich, and Temkin model plot for Cd(ii) and Pb(ii) adsorption on BC/MoS_2_/PAA composite

Isotherm model	Parameter	Pb(ii)	Cd(ii)
Langmuir	*q*_max_ (mg g^−1^)	26.47	8.23
	*K*_L_ (L mg^−1^)	1.22	3.26
	*R*_L_	0.052	0.029
	*R* ^2^	0.9832	0.9914
Freundlich	*K*_F_ (mg g^−1^) (L mg^−1^)^1/*n*^	14.63	5.82
	1/*n*	0.266	0.437
	*R* ^2^	0.6508	0.9232
Temkin	*b*_T_ (kJ mol^−1^)	0.47	1.59
	*K*_T_ (L g^−1^)	15.52	47.88
	*R* ^2^	0.7341	0.9832

### 3.7. Effect of co-existing ion and regeneration test

**Fig. 10:**
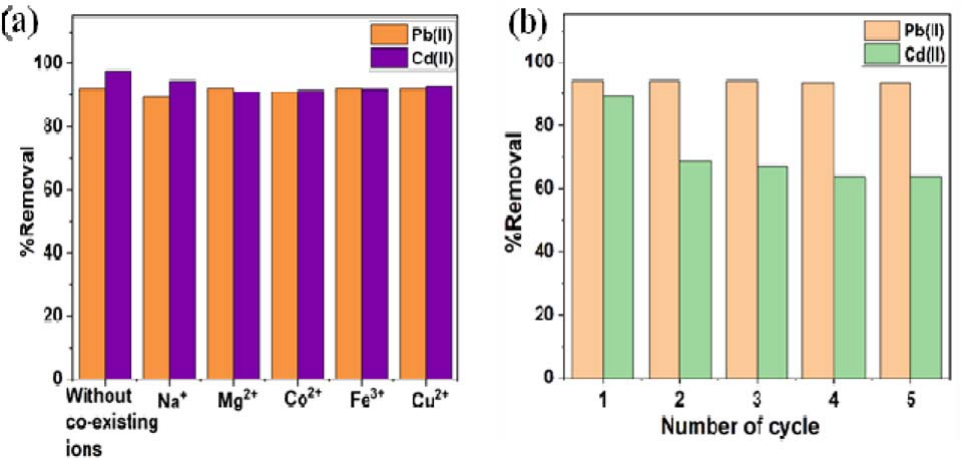
(a) Influence of co-existing ions on Pb(ii) and Cd(ii) adsorption using BC/MoS_2_/PAA composite (b) regeneration test of BC/MoS_2_/PAA composite towards Pb(ii) and Cd(ii) after five regeneration cycles.

Various inorganic metal cations present in environmental wastewater, in competition, may occupy the adsorption sites on the adsorbent thereby affecting the removal efficiency of Cd(ii) and Pb(ii). To investigate the possibility of BC/MoS_2_/PAA application in wastewater, the influence of Na(ii), Mg(ii), Co(ii), Fe(iii) Mg(ii), and Cu(ii) was evaluated to study the impact on removal of Cd(ii) and Pb(ii) as shown in Fig. 10a. The removal efficiency for Pb(ii) and Cd(ii) on BC/MoS_2_/PAA in the absence of co-existing ions were 92.04% and 97.42%, respectively. For Pb(ii), no significant impact on the adsorption efficiency in presence of co-existing cations, suggesting that BC/MoS_2_/PAA composite exhibited high a selectivity for Pb(ii). The selectivity order trend was Pb(ii) > Mg(ii) > Fe(iii) > Cu(ii) > Co(ii) > Na(i). For Cd(ii), the selectivity order trend was Cd(ii) > Na(i) > Cu(ii) > Fe(iii) > Co(ii) > Mg(ii), showing that Fe(iii), Co(ii) and Mg(ii) have some slight impact on the adsorption efficiency, possibly due to difference in ionic radius of each metal ion. Elements such as Fe(iii) and Mg(ii) with smaller ionic radius of 0.55 Å and 0.72 Å tend to occupy the adsorption site on BC/MoS_2_/PAA composite rapidly compared to Cd(ii) with a larger ionic radius (0.74 pm), which get saturated rapidly due to steric hindrance on the BC/MoS_2_/PAA surface, thereby leading to lower adsorption in the presence of Fe(iii) and Mg(ii) as co-existing ions^50^ while the impact of Co(ii) on the adsorption of Cd(ii) is due to difference in electronegativity (Co^2+^ (1.88) and Cd^2+^ (1.69)). However, the superior selectivity towards Pb(ii) than Cd(ii) can be principally ascribed to the difference in electronegativity and strong soft–soft interactions according to Pearson hard soft acid base (HSAB) theory,^51^ in which the sulfide S^2−^ sites on the BC/MoS_2_/PAA surface are considered as soft bases, which preferred to interact strongly with softer acids, thereby forming metal–sulfur complexes. The Pb(ii) and Cd(ii) are regarded as acids, which could be quantified based on the relationship of absolute hardness (*η*) values. The trend of absolute hardness is followed by Pb (8.46 eV) < Cd (10.29 eV),^52,53^ indicating that Pb(ii) is softer than Cd(ii), suggesting high selectivity for Pb(ii) than Cd(ii), which further supports the practical utilization of this advanced material.^54^

Considering the economic cost of wastewater treatment and disastrous impact of secondary pollution, hence, there is need to understand the stability of the BC/MoS_2_/PAA therefore, regeneration studies are very vital. In the desorption experiment, 0.1 M of HNO_3_ (10 mL) was used as a suitable eluent for the reusability study in five successive cycles at optimized conditions. The BC/MoS_2_/PAA loaded with Pb(ii) and Cd(ii) was added to 10 mL of 0.1 M HNO_3_ solution at optimized condition, then washed with water several times, dried, and reused in the next cycles. As seen in Fig. 10b, the percentage removal of the BC/MoS_2_/PAA composite for Cd(ii) and Pb(ii) in five successive cycles exhibited excellent adsorption performance, indicating that the BC/MoS_2_/PAA composite could be effectively regenerated. For Pb(ii), the BC/MoS_2_/PAA composite shows a high stability after five cycles as no significant decrease in removal efficiency was observed from cycle 1 (92.19%) to cycle 5 (91.65%), while for Cd(ii), the percentage removal in cycles 1 and 2 drastically reduced from 90.07% to 68.59% and remain almost steady from cycle 2 to cycle 5. The decrease in the removal percentage might be due to the incomplete desorption and the loss of adsorbents in the separation process.^47^ Hence, the regeneration efficiency was quantified over five adsorption–desorption cycles, and the adsorbent retained (∼91% and ∼60% for Pb(ii) and Cd(ii)) of its original capacity after five cycles, indicating good reusability and reduced environmental burden. In general, the adsorption performance and selectivity exhibited by the synthesized BC/MoS_2_/PAA composite show its capability as a potential composite material for wastewater treatment.

The stability of the BC/MoS_2_/PAA composite was assessed by leaching tests, which measured the release of molybdenum and sulfur during the adsorption of Pb(ii) and Cd(ii) under optimized conditions. The results indicated negligible leaching of both molybdenum and sulfur (see SI, Table S2) when in the presence of Cd(ii) and Pb(ii), compared to the total mass of Mo and S incorporated during the synthesis of the BC/MoS_2_/PAA composite. This suggests that the composite exhibits high stability and is an effective and durable adsorbent for wastewater treatment. Consequently, the adsorption capacity for Pd(ii) and Cd(ii) on the BC/MoS_2_/PAA composite (Table 3) was found to be superior to that of other commonly used adsorbents.

**Table 3:** Comparison of the maximum adsorption capacities (*q*_max_) for Cd(ii) and Pb(ii) adsorption onto different adsorbents

Adsorbate	Adsorbent	pH	*q*_max_ (mg g^−1^)	Ref.
Pb(ii)	Rice husk ash	5.82	12.61	55
	Pinewood biochar	5.0	4.25	56
	Modified MWCNTs	4	22.10	57
	Activated carbon	5	21.80	58
	BC/MoS_2_/PAA	6	26.47	This study
Cd(ii)	Chitin-based Chitorem SC-80	6	1.81	59
	Ca-bentonite	6	7.28	60
	Low grade-phosphate	5	7.54	61
	Bagasse fly ash	6	2.00	62
	BC/MoS_2_/PAA	7	8.23	This study

### 3.8. Analytical performance

To evaluate the analytical merit of the proposed method, the standard solution of Pb(ii) and Cd(ii) ion with different concentration were measured using ICP-OES. The calibration curves were linear in the range of 1 to 30 mg L^−1^ for Pb(ii) and 0.5 to 20 mg L^−1^ for Cd(ii). The limit of detection (LOD) was calculated as 2.35 mg L^−1^ and 0.80 mg L^−1^ for Pb(ii) and Cd(ii), respectively.

The cost of adsorbent preparation can be determined based on the cost of raw materials and electricity used for the synthesis of BC/MoS_2_/PAA composite, the cost estimate was 50.69 Thailand baht compared to commercial activated carbon (Sigma Aldrich) which 80.34 Thailand baht as shown in Table S3 (see SI).

### 3.9. Reaction mechanisms

**Fig. 11:**
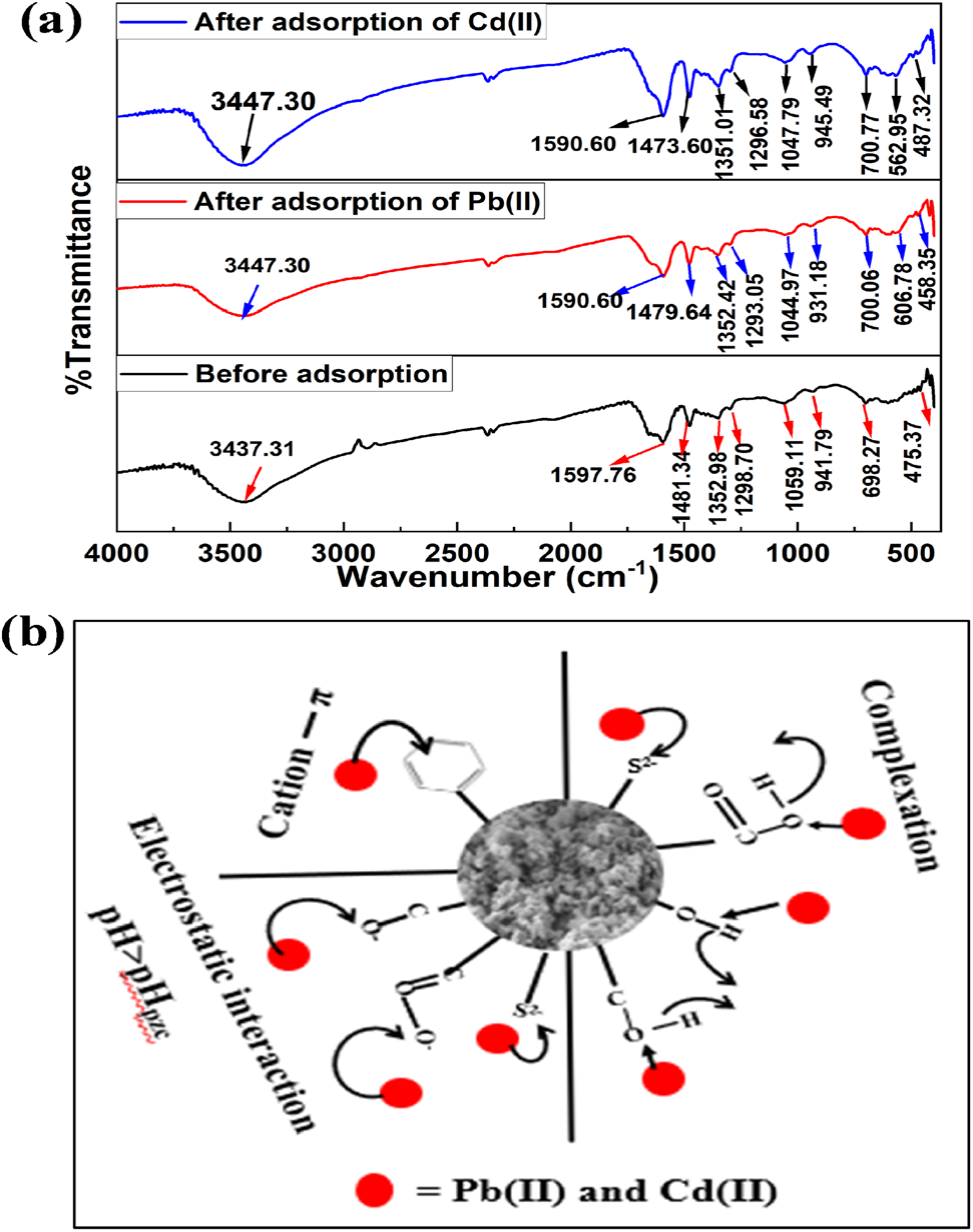
(a) FTIR spectra of before and after adsorption of Pb(ii) and Cd(ii) (b) possible sorption mechanism on BC/MoS_2_/PAA composite for Pb(ii) and Cd(ii).

In order to explore the adsorption mechanism of Cd(ii) and Pb(ii) as shown in Fig. 11, the negatively charged surface of the BC/MoS_2_/PAA composite (Fig. 1) as confirmed by the pH of point of zero charge (pH_pzc_), which favored the electrostatic interaction between the positively charged heavy metals (Cd(ii) and Pb(ii)) and the negatively charged surface of BC/MoS_2_/PAA composite.^47^ Inner layer adsorption of Cd(ii) and Pb(ii) on BC/MoS_2_/PAA composite can be attributed to the formation of Pb–S and Cd–S complex based on softness as explained by the HSAB theory,^63^ where the Cd(ii) and Pb(ii) is a soft acid that has a high affinity for sulfur (S^2−^) atom (soft base) present on the MoS_2_ in the aqueous solution through complexation and electrostatic interactions, which is considered to be plausible mechanistic approach.^64^ In Fig. 11a, the FTIR result after adsorption of Cd(ii) and Pb(ii) were shown. After adsorption of Cd(ii) and Pb(ii), the stretching peak of hydroxyl group shifted from 3437.31 to 3447.30 cm^−1^ and become more intense for after Cd(ii) adsorption, suggesting that complexation take place *via* complexation. The peak vibration at 1590.60 cm^−1^ and 931.18 cm^−1^ for Pb(ii) and 1590.60 cm^−1^ and 945.49 cm^−1^ for Cd(ii) are attributed to benzene ring C=C group and aromatic C–H stretching, indicating Pb(ii)–π and Cd(ii)–π interaction.^65^

The peaks at 1479.64 cm^−1^, 1352.42 cm^−1^, and 1044.97 cm^−1^ for Pb(ii) and 1473.60, 1351.01, and 1047.79 cm^−1^ for Cd(ii) are attributed to phenolic hydroxyl or –COO– and C–O, suggesting that the oxygen-containing group are involved in complex formation with Cd(ii) and Pb(ii) during the sorption process.^65^ Finally, new peak at 606.78 and 562.95 cm^−1^, indicate the formation Pb–S and Cd–S complex *via* complexation. As showed in Fig. 11b, complexation *via* oxygen-containing group, electrostatic interaction, and cation–π bonding are possible adsorption mechanism that took part in the sorption of Cd(ii) and Pb(ii) on BC/MoS_2_/PAA composite.

## 4. Conclusion

In this study, a heterojunction material, BC/MoS_2_, was synthesized by modifying biochar (BC) derived from coconut shell with MoS_2_ and poly(acrylic acid) (PAA) through a simple one-step hydrothermal process to obtain the BC/MoS_2_/PAA composite. The incorporation of PAA played a crucial role in enhancing the structural properties of the composite, promoting the formation of well-layered BC with a uniform dispersion of MoS_2_, resulting in increased interlayer spacing. The structural characteristics of the BC/MoS_2_/PAA composite were confirmed through FT-IR, XRD, SEM-EDS, BET, and Raman analyses, which also demonstrated its highly effective adsorption performance for Pb(ii) and Cd(ii). The maximum adsorption capacities for Pb(ii) and Cd(ii) were 26.47 mg g^−1^ and 8.23 mg g^−1^, respectively, within 30 minutes. The adsorption kinetics followed a pseudo-second-order model for both metals. Additionally, the adsorption isotherms were well-described by the Langmuir isotherm model, indicating monolayer adsorption on a homogeneous surface. The BC/MoS_2_/PAA composite exhibited good selectivity for Pb(ii) and Cd(ii) in the presence of competing ions and demonstrated high reutilization potential. FTIR analysis after adsorption indicated that the adsorption mechanisms were primarily governed by electrostatic interactions, surface complexation, and cation–π interactions, facilitated by the presence of oxygenated functional groups, S^2−^, and π–π bonding in the composite. Overall, this composite offers a promising alternative material for wastewater remediation, being both economically viable and environmentally friendly, with significant potential for the removal of Cd(ii) and Pb(ii).

## Author contributions

S. H. O.: methodology, visualization, investigation, formal analysis, validation, writing – original draft, writing – review & editing. W. A.: formal analysis, validation, writing – original draft, writing – review & editing. I. A. D.: funding acquisition, writing – review & editing. M. A.: supervision, methodology, resources, writing – review & editing. R. J. D.: resources, writing – review & editing. C. K.: supervision, methodology, resources, funding acquisition, writing – review & editing, project administration.

## Conflicts of interest

The authors declare that they have no known competing financial interests or personal relationships that could have appeared to influence the work reported in this paper.

## Supplementary Material

RA-015-D5RA03405A-s001

## Data Availability

The materials will be provided upon request, subject to availability. Additional figures and tables are available in the supplementary file that are necessary to complete this work. See DOI: https://doi.org/10.1039/d5ra03405a.
