# Supplementary material for: Synthesis of biochar/MoS2 composite modified with poly(acrylic acid) (BC/MoS2/PAA) for the removal of Cd(ii) and Pb(ii) from wastewater
Source: RSC Adv. 2025 Aug 26;15(37):30123–34. doi: 10.1039/d5ra03405a (PMC12378402; doi:10.1039/d5ra03405a)
Supplement: RA-015-D5RA03405A-s001 [file RA-015-D5RA03405A-s001.pdf]

Supplementary Information (SI) for RSC Advances.
This journal is © The Royal Society of Chemistry 2025

Supplementary Materials

Synthesis of Biochar/MoS_2 Composite Modified with Poly (acrylic acid) (BC/MoS_2/PAA) for the removal of Pb(II) and Cd(II) from Wastewater

Salami Hammed Olawale [a,b], Waleed Alahmad [a], Ibrahim A. Darwish [c], Mohammad Ashfaq [d], Ryhan J. Darling [e], Charoenkwan Kraiya [a,b,*]

[a] Department of Chemistry, Faculty of Science, Chulalongkorn University, Bangkok 10330, Thailand.

[b] Electrochemistry and Optical Spectroscopy Center of Excellence, Department of Chemistry, Faculty of Science, Chulalongkorn University, Bangkok 10330, Thailand.

[c] Department of Pharmaceutical Chemistry, College of Pharmacy, King Saud University, P.O. Box 2457, Riyadh 11451, Saudi Arabia.

[d] Department of Biotechnology, School of Science, Woxsen University, Hyderabad, 502345, Telangana, India.

[e] Department of Biochemistry, Medical College of Wisconsin, Milwaukee, WI 53226, USA.

* Corresponding author: mohdashfaqbiotech@gmail.com, charoenkwan.k@chula.ac.th

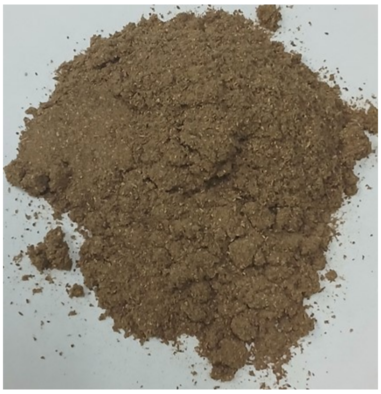

Powdered coconut shell

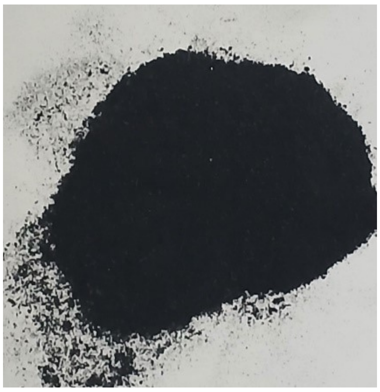

Biochar (BC)

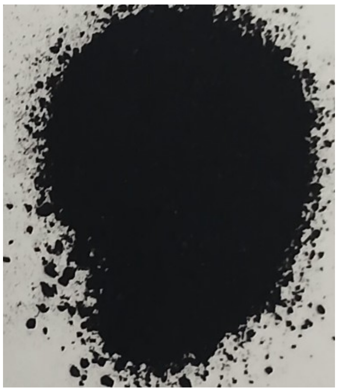

BC/MoS_2/PAA

Fig. S1. Photograph of synthesize BC and BC/MoS_2/PAA composite

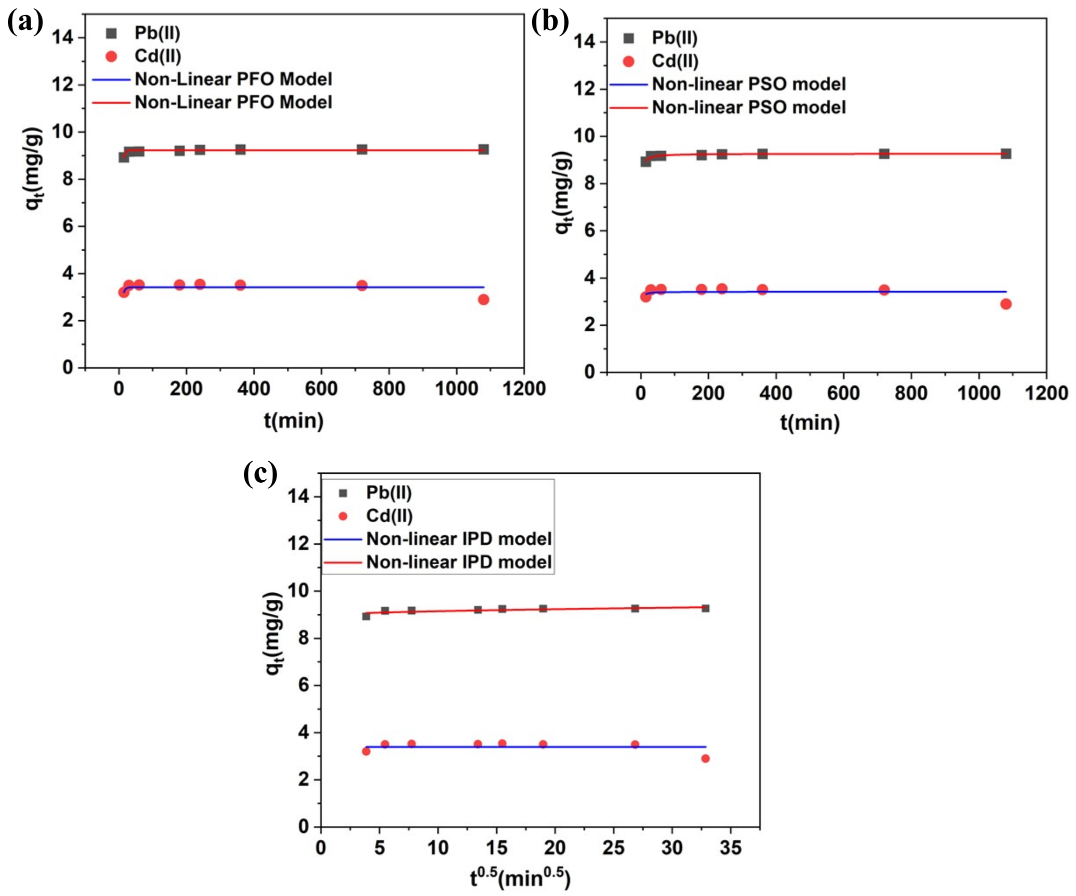

Fig. S2. Non-linear adsorption kinetic model analysis. (a) Pseudo-first order (PFO) (b) Pseudo-second order (PSO) (c) Intraparticle diffusion model (IPD) for Pb(II) and Cd(II). (Initial concentration: 10 mg/L; pH 6 for Pb(II); pH 7 for Cd(II); adsorbent dosage = 0.010 g for Pb(II), 0.025 g for Cd(II); volume = 10 mL) onto BC/MoS_2/PAA)**.**

Table S1. Kinetic models, non-linear equations for adsorption of Pb(II) and Cd(II) onto $BC/MoS_2/PAA$ composite

Model	Non-linear equation
Pseudo-first order (PFO)	$q_t = q_e(1-e^{-k_1 t})$
Pseudo-second order (PSO)	$q_t = \frac{q_e^2 k_2 t}{q_e k_2 t + 1}$
Intra-particle diffusion (IPD)	$q_t = k_{id} t^{1/2} + C$

Where q_e and q_t are the adsorbed amount at the equilibrium and time t, respectively (mg/g); k_1 and k_2 are the pseudo-first-order rate constant (min^{-1}) and pseudo-second-order rate constant for the adsorption process (mg/g min); k_{id} is the intraparticle diffusion rate constant (mg/g $min^{0.5}$); and *C* is a constant and proportional to boundary layer thickness, mg/g, q_{max} (mg/g) is the maximum adsorption capacity.

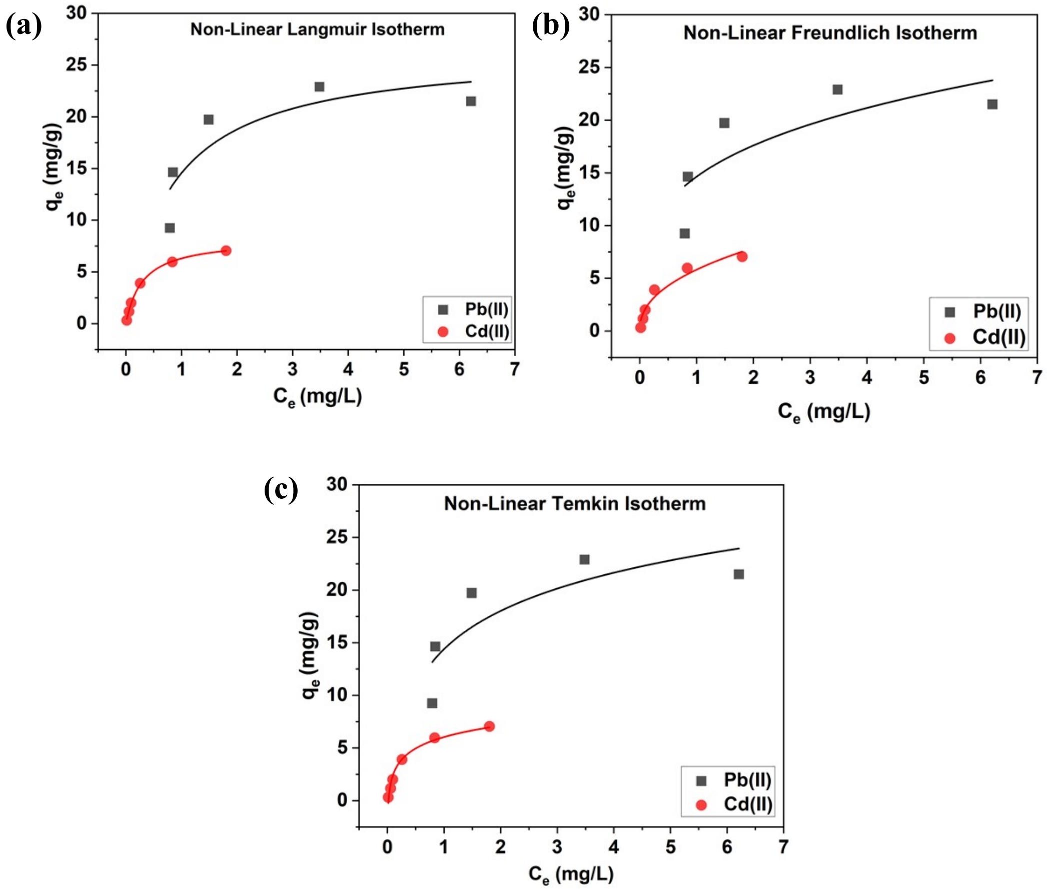

Fig. S3. Non-linear adsorption isotherm model analysis. (a) Langmuir (b) Freundlich (c) Temkin for Pb(II) and Cd(II). (Initial concentration: 10 mg/L; pH 6 for Pb(II); pH 7 for Cd(II); adsorbent dosage = 0.010 g for Pb(II), 0.025 g for Cd(II); volume = 10 mL) onto BC/MoS_2/PAA)**.**

Table S2. The leaching concentrations of Mo and S during adsorption of Pb(II) and Cd(II) onto BC/MoS_2/PAA adsorbent at optimized conditions (Volume= 10 mL; pH = 6, dose = 0.010 g for Pb(II) and pH = 7, dose = 0.025 g for and Cd(II); contact time = 30 min for Pb(II) and Cd(II) and initial concentration =10 mg/L for Pb(II) and Cd(II)).

	Concentration, mg/L	
Metals / Adsorbate	Pb(II)	Cd(II)
Mo	0.63±0.0023	3.4±0.012
S	3.39±0.035	1.68±0.0052

Table S3. Cost-analysis of 1 g prepared BC/MoS_2/PAA composite and comparison with market price of activated carbon.

Raw materials	Amount	Unit	Cost (Baht)
Waste coconut shell	10	g	0.0
$(NH_4)_6Mo_7O_{24}.4H_2O$	0.575	g	3.66
Thiourea	1.15	g	0.0619
Polyacrylic acid	0.50	g	4.99
Distilled water	100	g	2.00
Electricity (pyrolysis, magnetic stirrer, ultrasonication and furnace)	1.0366	kWh	40.02
**Total cost**			**50.69**
Commercial activated carbon (Sigma Aldrich)	1	g	**80.34**
